# Recruitment of Cln3 Cyclin to Promoters Controls Cell Cycle Entry via Histone Deacetylase and Other Targets

**DOI:** 10.1371/journal.pbio.1000189

**Published:** 2009-09-08

**Authors:** Hongyin Wang, Lucas B. Carey, Ying Cai, Herman Wijnen, Bruce Futcher

**Affiliations:** 1 Department of Molecular Genetics and Microbiology, Stony Brook University, Stony Brook, New York, United States of America; 2 Graduate Program in Genetics, Stony Brook University, Stony Brook, New York, United States of America; 3 Graduate Program in Molecular and Cellular Biology, Stony Brook University, Stony Brook, New York, United States of America; Yale University, United States of America

## Abstract

In yeast, the G1 cyclin Cln3 promotes cell cycle entry by activating the transcription factor SBF. In mammals, there is a parallel system for cell cycle entry in which cyclin dependent kinase (CDK) activates transcription factor E2F/Dp. Here we show that Cln3 regulates SBF by at least two different pathways, one involving the repressive protein Whi5, and the second involving Stb1. The Rpd3 histone deacetylase complex is also involved. Cln3 binds to SBF at the *CLN2* promoter, and removes previously bound Whi5 and histone deacetylase. Adding extra copies of the SBF binding site to the cell delays Start, possibly by titrating Cln3. Since Rpd3 is the yeast ortholog of mammalian HDAC1, there is now a virtually complete analogy between the proteins regulating cell cycle entry in yeast (SBF, Cln3, Whi5 and Stb1, Rpd3) and mammals (E2F, Cyclin D, Rb, HDAC1). The cell may titrate Cln3 molecules against the number of SBF binding sites, and this could be the underlying basis of the size-control mechanism for Start.

## Abstract 2

In yeast, titration of an increasing number of molecules of the G1 cyclin Cln3 by a fixed number of DNA-bound molecules of the transcription factor SBF might underlie the dependence of cell cycle entry on cell size.

## Author Summary

Cells seem to divide only after they have grown “big enough.” Entry into the cell cycle, at a point called Start in budding yeast, is triggered by activation of the Cln3 cyclin-dependent kinase (CDK), which in turn activates downstream transcription. We find that the Cln3-CDK acts through a histone deacetylase, as well as through the previously discovered repressor Whi5, to activate the SBF transcription factor and trigger entry into the cell cycle. The system is strikingly similar to the one in mammalian cells, which relies on Cyclin D, CDK, the transcription factor E2F, its repressor Rb, and the histone deacetylase system. There is preliminary evidence that as the yeast cell grows in size, the increasing number of Cln3 molecules is titrated against the fixed number of Cln3-CDK-SBF binding sites in genomic DNA, and that this cell size-dependent titration could be the mechanism that makes cell cycle entry dependent on cell size.

## Introduction

The budding yeast *Saccharomyces cerevisiae* commits to cell-cycle entry at a point called “Start,” equivalent to the restriction point in animals. Start depends on cell growth to critical size [1]–[5]. At the molecular level, Start coincides with and depends on a Start-specific burst of transcription of over 100 genes including the G1 cyclins *CLN1* and *CLN2*, the S-phase cyclins *CLB5* and *CLB6*, and many genes for budding and DNA synthesis [6],[7]. The burst of transcription at Start depends on two closely related transcription factors SBF and MBF, each of which contains the transcriptional modulator Swi6, and a sequence-specific DNA binding protein, Swi4 in SBF, and Mbp1 in MBF [8]. SBF is most important for the transcription of *CLN1* and *CLN2*, and these G1 cyclins are most important for propelling the cell cycle forward. SBF is found bound to the *CLN1* and *CLN2* promoters in early G1, well before Start, but at this time does not induce any transcription [9]–[12]. Indeed, in early G1, SBF may repress transcription. When cells have grown to critical size, the SBF is somehow converted to a transcriptional activator, and induces transcription of many genes including *CLN1* and *CLN2*.

The G1 cyclin Cln3, in combination with the cyclin dependent kinase Cdc28 (or Cdk1), is a key regulator of Start [13],[14], and is critical for the size-dependent activation of SBF and MBF [7], converting them from their early G1 repressive forms into the late G1 activating forms. Consistent with the idea that *CLN3* is a critical activator of Start, hyperactive alleles of *CLN3* (e.g., *WHI1-1*), and over-expression of *CLN3*, accelerate Start to smaller cell sizes, whereas deletion of *CLN3* delays Start to much larger cell sizes [13],[14]. A *cln3* null mutant, despite having a delayed Start and large cells, is viable because there are alternative methods of inducing transcription of *CLN1* and *CLN2*. The most important alternative route depends on the mysterious gene *BCK2*. The mechanism by which the Bck2 protein activates *CLN1* and *CLN2* transcription is still largely unknown [15],[16]. A *cln3 bck2* mutant is inviable in most genetic backgrounds precisely because it does not express sufficient amounts of *CLN1* or *CLN2*, and inviability can be suppressed by the expression of *CLN2* from a heterologous promoter [17],[18].

An obvious model for the *CLN3*-dependent activation of SBF is that the Cln3-Cdc28 kinase complex might phosphorylate SBF, thus activating it. However, no evidence for this model has been found [19]. Instead, there has been an accumulation of evidence that Cln3 works, at least in part, by inhibiting a repressor of SBF. Costanzo et al. and de Bruin et al. have identified Whi5 as one such repressor [20],[21]. The Whi5 protein associates with SBF on the *CLN2* promoter to repress transcription, and Cln3-Cdc28 phosphorylates and antagonizes Whi5 [20],[21]. Furthermore, deletion of *WHI5*, like over-expression of *CLN3*, accelerates Start to smaller cell sizes [22], and the *whi5* null mutant suppresses the inviability of the *cln3 bck2* double mutant [20],[21].

Although Whi5 is clearly an important target of Cln3, and an important regulator of SBF, it may not be the only target. Costanzo et al. found some evidence that *whi5* null mutants were still responsive to *CLN3*, suggesting that *CLN3* was also acting by at least one alternative pathway.

An enduring mystery has been the link between cell size and the activation of SBF. Cln3-Cdc28 activates SBF only when cells have grown to a critical size. But Cln3-Cdc28 is present even in very small cells. At least in slowly growing G1 cells, Cln3 abundance increases through G1 as the cell grows more-or-less in proportion to cell size and total cell protein. That is, its absolute abundance increases, but its relative abundance (relative to cell volume, or relative to protein content) does not, or at least not by very much [7],[23]. How does a small increase in abundance trigger Start at a critical size? One possibility is that Cln3 is titrated against something that is constant per cell. Here, we suggest that increasing amounts of Cln3 are titrated directly against the SBF bindings sites in genomic DNA, which are of course constant in number through G1 phase. At a sufficiently high Cln3/SBF site ratio, SBF is activated, and Start ensues.

Finally, it is remarkable how well eukaryotic cell cycle control mechanisms have been conserved, with the functional replacement of fission yeast *cdc2* by human *cdc2* (*CDK1*) [24] an early and striking example. The yeast system for promoting Start is analogous and perhaps homologous to the mammalian system, with SBF, Cln3, and Whi5 playing roles similar to those of E2F-Dp, Cyclin D, and Rb, respectively [25]. Our results suggest that the analogy goes even deeper, with both the yeast and mammalian system making critical use of the Rpd3 histone deacetylase to repress transcription of S-phase genes.

## Results

### Whi5 Is Not the Sole Target of Cln3

The only known role of Cln3 is to activate SBF and MBF; evidence for this is that *swi6* mutants (which lack both SBF and MBF) are completely nonresponsive to *CLN3* [19]. That is, the cell size of *swi6* mutants is unaffected by over-expression or under-expression of *CLN3*. If Whi5 is the one and only target of Cln3, then the size of *whi5* mutant cells, like that of *swi6* mutant cells, should also be nonresponsive to *CLN3*. Whether or not this is true is unclear, although Costanzo et al. [20] found some evidence that *whi5* mutants did respond to *CLN3*.

**Figure 1:**
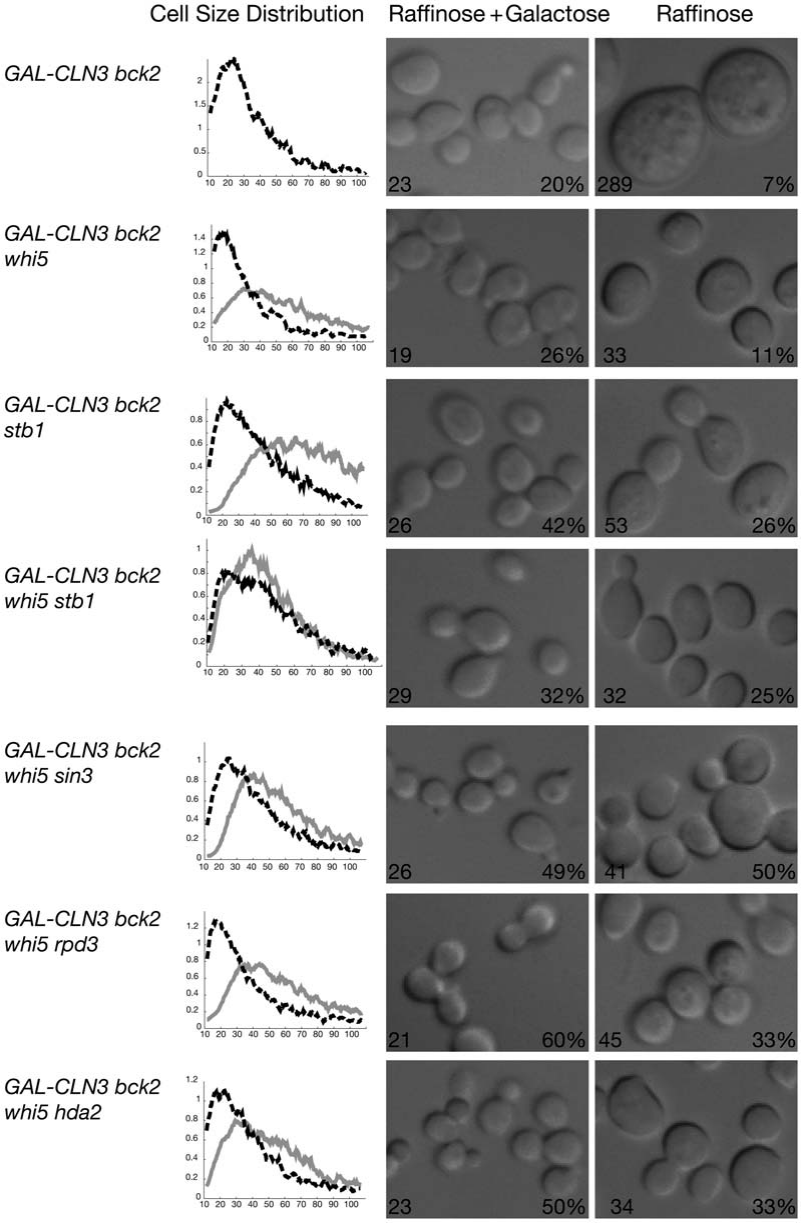
Responsiveness to *CLN3*. Various mutants containing *bck2* and *CLN3* under the control of the *GAL* promoter (*GAL-CLN3*) as the only allele of *CLN3* were grown in YEP raffinose medium (*CLN3* off, solid grey line), split into two aliquots, and galactose was added to one aliquot (*CLN3* on, dotted black line). Cell volume distributions were measured with a Coulter Channelyzer (left column), and photomicrographs were taken (right two columns). The median cell size (fl) as measured by the Coulter Channelyzer is shown in the bottom left corner of each photograph, and the percentage budding is shown in the bottom right corner. A shift to smaller cell sizes and to higher percent budding in galactose shows responsiveness to *CLN3*. The changes in budding are statistically significant at the *p*<0.01 level for all strains except the *whi5 stb1* strain (4th from top) and the *whi5 sin3* strain (5th from top). The cell size distribution is not shown for *GAL-CLN3 bck2* cells in raffinose (top panel) because these extremely large cells are off-scale. Strains used, from top to bottom, are: LC517, LC520, LC518, LC504, LC524, LC521, and LC523.

To address this issue in a more sensitive way, we used strains containing a *bck2* mutation. Since Bck2 is a redundant with Cln3 for expression of *CLN1*, *CLN2*, and other genes [17],[18], *bck2* mutants are even more sensitive than wild-type cells to the effects of *CLN3*. Thus we compared *bck2 WHI5* cells with *bck2 whi5* cells with respect to the effect of *CLN3* on cell size. Results (Figure 1, top two panels) show clearly that both genotypes are still responsive to *CLN3*. Thus, Cln3 can affect cell size, and presumably SBF/MBF activation, even in a *whi5* null strain, suggesting it has some target in addition to Whi5.

### A Screen for Additional Repressors of SBF

As described above, a *cln3 bck2* double mutant is inviable in many strains, because SBF cannot be activated, and so *CLN1*, *CLN2*, *CLB5*, and *CLB6* cannot be expressed at sufficient levels. However, a *whi5* mutation relieves some of the repression of SBF, and so a *cln3 bck2 whi5* mutant is viable.

Mutations in other putative repressors of SBF might also suppress the inviability of a *cln3 bck2* strain. Thus we constructed a *cln3 bck2* strain kept alive by plasmid-borne *MET-CLN2* (a construct where *CLN2* expression is repressed by methionine). This strain is viable in the absence of methionine, but dies with a G1 arrest in the presence of methionine. The strain was mutagenized using a transposon library (so that mutant genes could be identified), and spread on +met plates to select suppressors.

This screen yielded two classes of mutants irrelevant to our studies. First, there were a variety of mutants (many *cis*-acting) that derepressed the *MET* promoter, and thus mis-expressed the plasmid-borne *MET*-*CLN2*. Second, there were mutants that by one means or another increased the expression of the *RME1* gene, which encodes a transcription factor that, among other things, binds directly to the *CLN2* promoter and increases *CLN2* expression [26]. We identified these irrelevant mutants using secondary screens; they were not further analyzed.

The screen also yielded four complementation groups that may be of direct relevance: *chd1*, *hda2*, *pho23*, and *stb1*. The *chd1* and *stb1* mutations were obtained many times each, whereas *hda2* and *pho23* were obtained only once each. No *whi5* mutation was obtained, but subsequent examination of the mutagenic transposon library by PCR showed that this library did not contain even one disrupted copy of *WHI5*.

*CHD1* is CHromoDomain 1, a nucleosome remodeling factor containing a chromodomain, (which can mediate binding to histones bearing methylated lysines), a helicase domain, and a DNA binding domain [27]. It is a component of both the SAGA and SILK complexes [27]. It is a likely mediator of SBF activity, but its relevance will be considered in a separate report. Interestingly, Rb-binding protein 1 (RBP1), a mediator of E2F repression in mammalian cells, also contains a chromodomain.

*HDA2* is Histone DeAcetylase 2, a member of the Hda1 histone deacetylase complex [27]. Its function is poorly understood. Although we do not further consider Hda2 here, it could well be a repressor at the *CLN2* promoter.

*PHO23* encodes a component of the Rpd3 histone deacetylase complex [28]. The Rpd3 histone deacetylase is a major histone deacetylase activity in yeast [29],[30], and moreover is the yeast ortholog of mammalian HDAC1, the histone deacetylase that interacts with E2F and Rb.

Finally, *STB1* (Sin Three Binder 1) was originally isolated as an interactor with Sin3 [31], and Sin3 is a targeting subunit for the Rpd3 histone deacetylase [32]. Stb1 has also been isolated as a protein binding to the Swi6 component of SBF and MBF, and modulating transcription [33],[34]. Thus Stb1 could be a link between SBF and the Rpd3 histone deacetylase complex.

**Figure 2:**
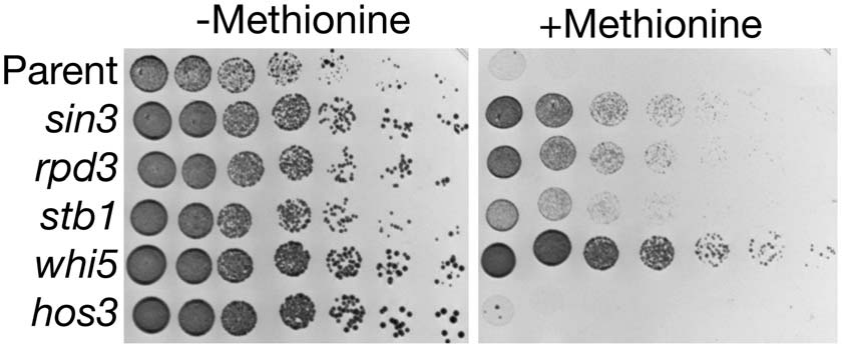
Suppressors of *cln3 bck2*. Various mutations were tested for ability to suppress lethality of a *cln3 bck2 rme1* {*MET-CLN2*} strain on +methionine medium. The parental strain (N497) is *cln3 bck2 rme1* {*MET-CLN2*}; other strains (top to bottom: N452, N453, N451, N499, and N454) have one additional mutation as indicated. Serial 4-fold dilutions were spread on −methionine (where *MET-CLN2* is expressed) or +methionine (where *MET-CLN*2 is repressed). The *HOS3* gene encodes a histone deacetylase, but this histone deacetylase was not identified in our suppressor screen and serves as a negative control.

The involvement of both Stb1 and Pho23 implicated the Rpd3 histone deacetylase complex at the *CLN2* promoter. Furthermore the Rpd3 complex has previously been implicated in the repression of various cell cycle genes, especially SBF or MBF dependent genes [35],[36]. Therefore we asked whether mutations in *RPD3* (encoding the catalytic subunit) or *SIN3* (encoding the targeting subunit) could, like mutations in *STB1*, *PHO23*, or *WHI5*, suppress the inviability of the *cln3 bck2* double mutant. Indeed, both *rpd3* and *sin3* did suppress the inviability of the *cln3 bck2* mutant (Figure 2). Consistent with this, D. Huang, S. Kaluarchchi, and B. Andrews (personal communication) have also found that *rpd3* can suppress the *cln3 bck2* double mutant. As judged by growth rate of the various mutants (Figure 2), *whi5* is the strongest suppressor.

Because *RME1* slightly activates *CLN2* transcription directly, and because one class of suppressor over-expressed *RME1*, we wondered about the relationship, if any, between *pho23*, *sin3*, *rpd3*, etc., and *RME1*. Therefore we analyzed the suppressors (*chd1*, *pho23*, *stb1*, *sin3*, *rpd3*, and *whi5*) in *cln3 bck2* strains that were either *RME1* or *rme1*. In the *RME1* background, all the suppressors could suppress inviability, and could lose the *MET-CLN2* plasmid. In the *rme1* background, the suppressors could again suppress inviability of the *cln3 bck2 MET-CLN2* strain on +met plates; however, strains of these genotypes could not lose the *MET-CLN2* plasmid (with the *whi5* strain being an exception, and able to lose the plasmid). This result suggested that the slight, residual expression from the repressed *MET-CLN2* construct was important for viability.

The inability of the suppressed *rme1* strains to lose the *MET-CLN2* plasmid meant there were two possible explanations for the suppression. First, it could be that some or all of the suppressors de-repressed the native, genomic *CLN2* locus, allowing viability, but that the degree of de-repressed *CLN2* expression was modest, and viability also required a trace of additional expression, which could come either from *RME1* (driving genomic *CLN2*), or from repressed *MET-CLN2* (expressing low, residual levels of *CLN2*). Second, it could be that some or all of the suppressors were activating *RME1* (thereby inducing native *CLN2*) and also de-repressing *MET-CLN2*.

**Figure 3:**
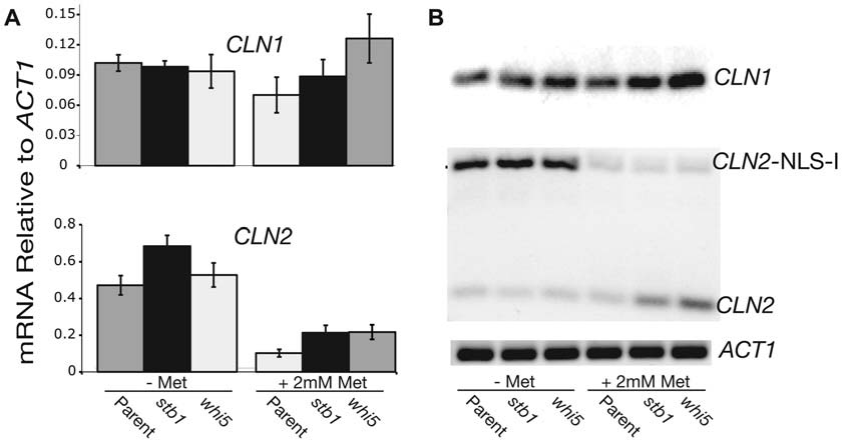
Analysis of *CLN1* and *CLN2* expression in the *cln3 bck2 stb1* and *cln3 bck2 whi5* strains. qPCR was used to measure the expression of *CLN1* and *CLN2* in two of the suppressor strains. The parental strain is *cln3 bck2 rme1* {*MET-CLN2*} (N497), and the two suppressor strains have, in addition, *stb1* (N451) or *whi5* (N499), as indicated. (A) The left panel shows quantitation of *CLN1* and *CLN2* mRNAs by qPCR, in the absence (−met) or presence (+met) of methionine. (B) The right panel shows the PCR products from (A) separated by agarose gel electorphoresis. The plasmid-borne allele of *CLN2* is *CLN2*-*NLS-I*; it carries a tag that increases the length of the mRNA and the PCR product, allowing expression of genomic *CLN2* to be distinguished from expression of plasmid-borne *MET-CLN2-NLS-I*. This demonstrates that the increased *CLN2* expression caused by *stb1* and *whi5* is specific for the genomic copy of *CLN2*. The qPCR measurement in (A) includes both forms of *CLN2*, suggesting that the increased expression of genomic *CLN2* in the *stb1* and *whi5* mutants is about 3-fold. The primers for amplification of *ACT1* flank the *ACT1* intron, yet only the mRNA-specific band was obtained, showing that no DNA was present in the RNA preparation. Error bars show the standard error of the mean.

Two lines of experimentation showed that the first possibility is correct. First, the transcript from the native *CLN2* locus differs in length from the *MET-CLN2* transcript. We used quantitative (q) PCR to show that the suppressors increase transcription of the native *CLN2* locus, but have no effect on transcription of *MET-CLN2* (Figure 3). The two strongest suppressors, *stb1* and *whi5*, activated *CLN2* transcription to similar extents (Figure 3). Expression of *CLN1* was also increased. Second, we integrated a second copy of *CLN2* at the *CLN2* locus, using a large restriction fragment that included sequences up to and including the flanking genes. The tested suppressors (*sin3*, *stb1*, and *whi5*) were able to suppress inviability of the resulting *cln3 bck2 rme1 2×CLN2* strain, and these strains were able to lose the *MET-CLN2* plasmid (unpublished data). Thus, in a 2×*CLN2* strain, neither *RME1* nor *MET-CLN2* is required for suppression; the suppressors must act by de-repressing the native *CLN2* locus.

These results establish that Stb1, Sin3, and Rpd3, like Whi5, play a role in the repression of SBF target genes. However, they do not establish whether Stb1, Sin3, and Rpd3 are additional components of the Whi5 pathway (i.e., Whi5 might act by attracting the Rpd3 complex), or whether some or all of these new repressors constitute the second pathway that allows *whi5* mutant cells to respond to *CLN3*. To address this, we did epistasis analysis. We constructed double mutants with *whi5* (i.e., *stb1 whi5*, *sin3 whi5*, *rpd3 whi5*), and asked whether any of these double mutants would reduce or eliminate responsiveness to *CLN3* (which would indicate that the new repressors are in the second new pathway). Unlike either of the single mutants, a *whi5 stb1* double mutant is almost nonresponsive to *CLN3* (Figure 1). Thus *STB1* likely defines a second pathway by which *CLN3* controls activity of SBF.

Epistasis analysis of *rpd3* and its targeting subunit *sin3* with *whi5* and with *stb1* gave complex results. The *whi5 sin3* and the *whi5 rpd3* mutants are still responsive to *CLN3* with respect to size (Figure 1, fifth and sixth panels), although the *whi5 sin3* mutant does not show any responsiveness with respect to budding. This suggests that *sin3* (in particular) and *rpd3* may be partially but not fully blocking the Stb1 pathway. But *stb1 sin3* double mutants are responsive to *CLN3* (unpublished data), suggesting that the *sin3* mutation is not fully blocking the Whi5 pathway. Previous experiments have established links between Sin3, Rpd3, and Stb1 [29],[31]. We feel there are several alternative interpretations of these data (see Discussion), the most likely being that Whi5, Stb1, and Swi6 all interact to some extent with the Rpd3 histone deacetylase complex. Consistent with this, Huang, Kaluarchchi and Andrews have recently found an association between Whi5 and Rpd3 by co-immunoprecipitation (personal communication).

### Rpd3, Sin3, and Stb1 Are Found at the *CLN2* Promoter, and Rpd3 and Sin3 Are Removed in a *CLN3*-Dependent Fashion

To further characterize the mechanisms by which Cln3 promotes transcription of SBF target genes, we used chromatin immunoprecipitation (ChIP) to build on the earlier work of Cosma, Nasmyth, and coworkers [9],[10] and observe events at the *CLN2* promoter (an important SBF target gene) as a function of Cln3 abundance. This is a challenging goal, since once cells have passed through Start, they repress transcription of SBF target genes by additional mechanisms [37]. Thus activation of SBF target genes under normal conditions is transient and difficult to characterize. Therefore, we constructed a strain with genotype *GAL-CLN3 bck2 cdc34-2* (i.e., *CLN3* is expressed from the *GAL* promoter). This strain can be synchronized in G1 before Start by growing in raffinose (i.e., without galactose, *CLN3* expression is off, its target G1 cyclins *CLN1* and *CLN2* are not expressed, and Start does not occur). When these G1 cells are then switched to raffinose plus galactose medium at 37°C (the restrictive temperature for the *cdc34-2* mutation), *CLN3* is turned on, SBF targets are transcribed, but progress through the cell cycle (and the consequent repression of SBF targets) does not occur because of the *cdc34-2* defect. Thus we can follow a cell population from a state where SBF genes are fully repressed in all cells (in raffinose medium) to a state where SBF genes are fully induced in all cells (in galactose medium at 37°C).

**Figure 4:**
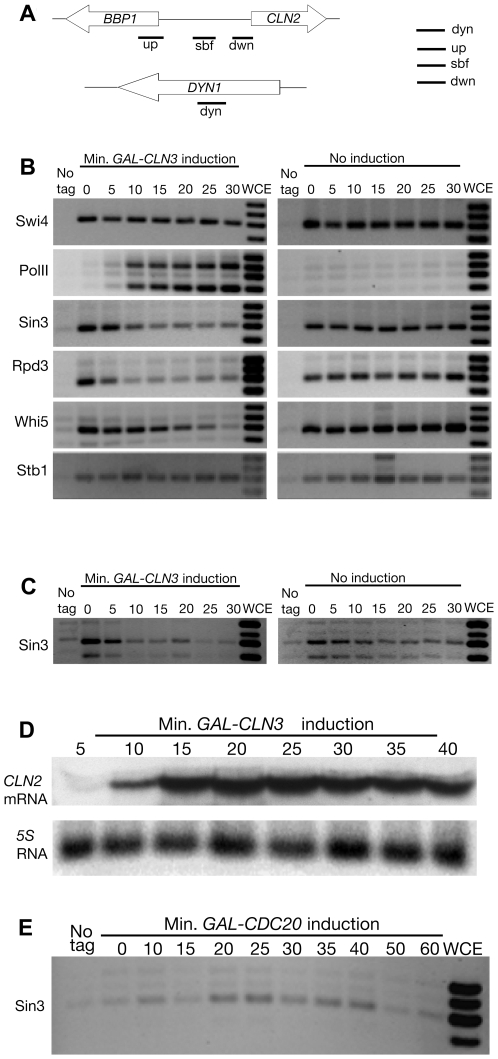
Proteins at the *CLN2* promoter. (A) Map of the *CLN2* promoter, showing the location of the up, sbf, and dwn probes in the *BBP1-CLN2* intergenic region. The sbf probe overlaps the consensus SBF binding sites. The dyn probe is in the middle of the *DYN1* open reading frame, several kilobases from the nearest promoter. On the right is shown the relative order of these probes on gels; i.e., the dyn probe has the lowest mobility, and the dwn probe the highest mobility. (B) Proteins at the *CLN2* promoter as a function of *CLN3* induction. *GAL-CLN3* expression was induced (left) or not (right) by the addition of galactose. Samples were taken from 0 to 30 min. Proteins were cross-linked to DNA and processed as described, and TAP-tagged proteins (from strains HWL61, HWL59, HWL51, HWL62, HWL77, and HWL128) were immunoprecipitated. Coprecipitated DNA fragments were identified by PCR using probes amplifying fragments described above in (A). Whole cell extract (WCE) was also amplified, as a positive control. Samples (HWL49) from cells lacking any TAP-tagged protein were processed as a negative control (No tag). The parental strain was HWL49, with partial genotype *GAL-CLN3 bck2 cdc34-2*. (C) As (B), but TAP-tagged Sin3 is being assayed by ChIP at the *YOX1* promoter, instead of at the *CLN2* promoter. (D) Kinetics of induction of the *CLN2* mRNA, by Northern analysis. 5S RNA serves as a loading control. (E) *GAL-CDC20 cdc20 SIN3-TAP* cells were arrested in M-phase in glucose, then *GAL-CDC20* was induced at 0 time with galactose medium. The amount of Sin3-TAP associated with the *CLN2* promoter was assayed during a timecourse. Budding initiates at about 50 min.

Figure 4 shows the fate of some relevant proteins at the *CLN2* promoter as a function of *CLN3* expression. As expected from previous work, Swi4 is at the SBF binding sites of the *CLN2* promoter at all times [10]–[12], regardless of the presence or absence of *CLN3*. RNA polymerase II is initially absent, but is recruited to the TATA box of *CLN2* (and to the TATA box of *BBP1*, the divergently transcribed, SBF-controlled gene at the other end of the intergenic region) 5 to 10 min after induction of *CLN3*. Northern analysis shows that the production of *CLN2* mRNA almost exactly coincides with recruitment of RNA pol II (Figure 4). Previous studies have shown that this recruitment of RNA pol II depends on Cdc28 kinase [9]. Stb1 is also found near the SBF binding sites, and its presence is not affected by induction of Cln3. Finally, Whi5, Sin3, and Rpd3 are all initially present near the SBF binding sites on the *CLN2* promoter, and each of these proteins is lost after *CLN3* is induced. Consistent with the relative early and *CLN3*-dependent loss of Rpd3, Huang, Kaluarchchi and Andrews have recently found that *CLN3* can reduce the amount of the Whi5-Rpd3 complex seen by co-immunoprecipitation (personal communication).

Several of these results were also repeated, with the same results, on the *YOX1* promoter, which is also regulated by *CLN3* and is coregulated with *CLN2* (Figure 4C).

Surprisingly, the loss of the repressive proteins is not obvious until 5 to 15 min (for Sin3 and Rpd3), or 15 to 25 min (for Whi5) after *CLN3* induction; that is, recruitment of RNA pol II, and the appearance of *CLN2* transcript, occur before all the repressive proteins are lost. There are at least three nonexclusive explanations for these kinetics: first, it could be that the repressive proteins are quickly phosphorylated and thereby inactivated as repressors by the Cln3-Cdc28 kinase complex; loss of the proteins from the promoter could be a secondary event. Second, it could be that Cln3-Cdc28 is promoting some positive event that directly induces transcription, and this precedes full loss of the repressive activities. Perhaps phosphorylation of Stb1 or Swi4 or Swi6, for instance, could directly promote transcription even in the presence of repressors. Third, there could be some systematic bias in our ChIP assay such that it is easier to see new proteins arriving at *CLN2* than to see old proteins leaving. Additional experiments will be required to distinguish these possibilities.

The *GAL-CLN3 bck2 cdc34-2* strain used in the experiments above allowed us to look at events at the *CLN2* promoter in a way that is powerful and sensitive, but also contrived. Therefore, we repeated some of the experiments in a different genetic background. We used a strain carrying a *cdc20* mutation and a galactose inducible *CDC20* gene (*GAL-CDC20*) to arrest cells at the *cdc20* block (mitosis; pre-anaphase), then release them synchronously. Although this approach is less sensitive than the *GAL-CLN3 bck2 cdc34-2* method, we were able to reproduce several of the main results. For example, Figure 4E shows Sin3 being recruited to the *CLN2* promoter early in G1, then leaving as cells exit G1 (in this experiment, budding begins at about 50 min).

**Figure 5:**
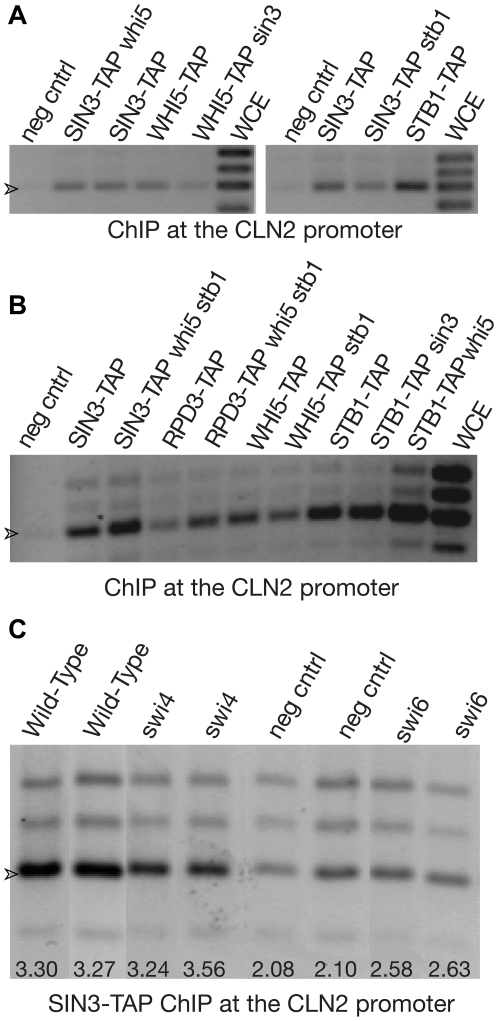
Dependency analysis. (A) ChIP analysis of proteins at the *CLN2* promoter in various mutants. Cells (left panel: S288c, HWL99, HWL110, OBS1, and HWL117; right panel S288c, HWL110, HWL119, and OBS5) were *CLN3 BCK2 CDC34* in exponential growth. The TAP-tagged protein being assayed is indicated, as is any additional mutation in the strain. The arrowhead (>) indicates the band containing the SBF binding sites. Other bands are as drawn in Figure 4A. (B) ChIP analysis of proteins at the *CLN2* promoter in various mutants. As in Figure 4A, but with a different selection of mutants. The arrowhead indicates the band containing the SBF binding sites. (C) ChIP analysis of Sin3-TAP at the *CLN2* promoter in wild-type, *swi4*, *swi6*, and untagged (negative control) strains. Ten independent experiments were done for each of the four genotypes. Results for all 40 experiments were obtained and tested statistically after “blinding” the samples (Table 1). The two median-most experiments for each of the four genotypes are shown here. The ratio of the intensity of the SBF band (arrowhead) to the sum of the intensities of the upper two bands (“dyn” plus “up”) is shown at the bottom of each gel lane.

We began to characterize the binding dependencies of some of the proteins at the *CLN2* promoter (Figure 5; Table 1); one obvious question is whether binding of the Sin3-Rpd3 complex depends on Whi5 or Stb1. Sin3 still binds to the *CLN2* promoter in a *whi5* mutant and also in an *stb1* mutant, and also in a *whi5 stb1* double mutant. To see if Sin3 binding was SBF dependent, we used both *swi4* and *swi6* mutations, and found that the association of Sin3 with the *CLN2* promoter is dependent on *SWI6* (*p* = 4×10^−3^), but only slightly if at all dependent on *SWI4* (Figure 5; Table 1). Presumably in the absence of Swi4, MBF (Mbp1+Swi6) is binding to the *CLN2* promoter and recruiting Sin3-Rpd3. The dependence of Rpd3 binding on Swi6 correlates with previous findings that Swi6 contains Cln3-modulated repressive domains [19]; these could be the regions responsible (directly or indirectly) for recruiting the Rpd3 complex. Stb1 and perhaps Whi5 could further influence the recruitment or activity of the Sin3-Rpd3 complex.

**Table 1:** Binding of Sin3 to the *CLN2* promoter in *swi4* and *swi6* mutants: *p*-values.

Strain	WT	*swi4*	*swi6*	Negative Control
WT		0.5	0.004	0.0003
*swi4*			0.0008	0.00001
*swi6*				0.1

*p*-Values for a test of a difference of means for ChIP analysis of the *CLN2* promoter fragment containing the SBF sites in strains containing tagged Sin3. The strains are wild-type (WT), *swi4*, and *swi6* strains all carrying tagged *SIN3*, and an untagged *SIN3* strain as a control. The experiment was done ten independent times in each strain. For each experiment, PCR-amplified DNA from ChIP was placed in a tube with a coded label by one investigator (HYW), and given to a second investigator (LBC). PCR fragments were separated by gel electrophoresis, and the ratio of the “sbf” band to the mean of the “up” and “dyn” bands (see Figures 4 and 5) was determined using image analysis software. Once ratios were determined, the label code was broken, and ratios were assigned to their genotypes. One-tailed *t*-tests were done to calculate *p*-values of the differences of the means of the logarithms of the ratios (Log ratios were used so as to produce a normal distribution). These *p*-values are shown above. For example, Sin3 does not appear to ChIP to the *CLN2* promoter in a *swi6* mutant, and the difference between the *swi6* mutant and the wild-type in this regard has a *p*-value of 4×10^−3^. Two typical (median) experiments were chosen from each of the ten sets of experiments and shown in Figure 5.

Robert et al. [35] previously found that Rpd3 associates with the promoters of *CLB6* and *PCL1*, which are regulated by SBF and by MBF. In contrast to our finding of Swi6 but not Swi4 dependence at the *CLN2* promoter, Robert et al. found that the association of Rpd3 with *CLB6* and *PCL1* required both Swi6 and Swi4. The reason for the difference between the studies with respect to the requirement for Swi4 is unclear, but in any case both studies agree that SBF is involved in the recruitment of the Rpd3 complex.

### Cln3 Is Found at the *CLN2* Promoter

**Figure 6:**
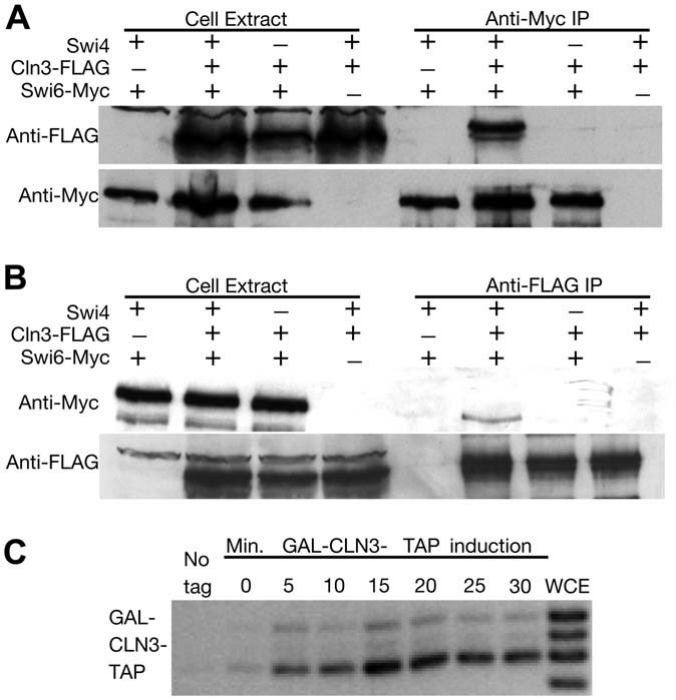
Cln3 associates with SBF and ChIPs to the *CLN2* promoter. (A) Cln3 co-immunoprecipitates with Swi6. Strains (HWL72, HWL112, HWL130) with various combinations of *CLN3* or *CLN3-FLAG* (pCM273), *SWI6* or *SWI6-Myc*, or *SWI4* or *swi4*, were grown and extracts made. In the left half of (A, these extracts were tested for the presence of the FLAG-tagged Cln3 and the Myc-tagged Swi6 by Western blotting with Anti-FLAG or Anti-Myc antibody). In the right half, proteins were immunoprecipitated with Anti-Myc antibody (directed against Swi6-Myc), and then these immunoprecipitates were tested by Western blotting for the presence of Swi6-Myc and Cln3-FLAG. (B) As 6A, except that the immunoprecipitation is done with the anti-FLAG antibody, followed by Western analysis with the anti-Myc antibody. (C) A ChIP experiment as in Figure 4B, for association of Cln3-TAP (HWL49, HWL63) with the *CLN2* promoter.

While it is clear that that Cln3-Cdc28 protein kinase complex somehow promotes the loss of Sin3, Rpd3, and Whi5 from the *CLN2* promoter, it is not clear how directly Cln3 acts, or exactly what proteins the Cln3-Cdc28 complex phosphorylates. We found that Cln3 co-immunoprecipitates with the Swi6 component of SBF, and that this co-immunoprecipitation depends on Swi4 (Figure 6). Thus, there is a relatively direct interaction between Cln3 and SBF. ChIP showed that Cln3 is found on the *CLN2* promoter close to the SBF binding sites (Figure 6), the same location as SBF, Whi5, Sin3, Stb1, and Rpd3. Cln3-Cdc28 is thus in a location suitable for the direct phosphorylation of these and other associated proteins. Both Whi5 and Stb1 have a very high density of consensus phosphorylation sites for the Cdc28 kinase (2.7 or 2.6 consensus and near-consensus sites per 100 amino acids, respectively), and previous work suggests that they are very likely substrates for Cln-Cdc28 kinase [20],[21],[34]. Swi6 and Swi4 also have multiple potential Cdc28 phosphorylation sites, and could be Cln3-Cdc28 substrates in vivo.

### Cln3 May Be Titrated by SBF and Its Binding Sites

The fact that Cln3 is at the *CLN2* promoter raises another issue. Yeast has 100 to 200 genes under the control of SBF and the related transcription factor MBF, and these genes typically have two, three, or more SBF/MBF binding sites each. Thus the total number of functional SBF and MBF binding sites in the cell is in the vicinity of 400. But the average number of Cln3 molecules in a haploid cell is only about 100 [38]. Of course there is considerable uncertainty in these measurements, but nevertheless it is likely that cellular SBF/MBF binding sites are in excess over Cln3.

This excess of binding sites could provide a basis for the critical size requirement for Start. As cells grow in mass, ribosome content, and protein synthetic capacity, they contain increasing numbers of Cln3 molecules [7]. Indeed, growth in the number of Cln3 molecules may be faster than the growth in mass [39]. Yet the number of SBF binding sites is fixed by DNA content. Thus, as the cell grows, it could titrate an increasing number of Cln3 molecules against a fixed number of SBF binding sites, which are initially in excess. At some ratio, the bound Cln3 could activate SBF, resulting in Start.

**Figure 7:**
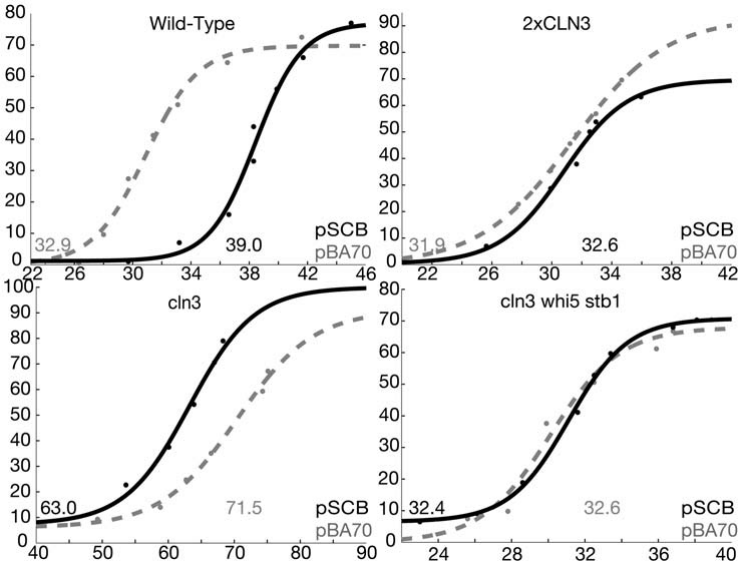
Cln3 may be titrated by SBF binding sites. Cells (BF305-15d, HWL148, HWL145, and HWL146) were transformed with an empty vector (pBA70, grey line) or the same vector carrying an insert with four tandem copies of an SBF binding site (“pSCB”) (pMT3579, black line). Cells were grown to early log phase, and fractionated by centrifugal elutriation. Fractions containing small unbudded cells with a mode volume of about 20 fl were chosen and re-inoculated. Cell size (*x*-axis, in fl) and budding (*y*-axis, percent) were followed with time. The median experiment of five total wild-type experiments is shown in the upper left. “Critical size” was defined to be the size (in fl) at which 50% of the cells became budded. This critical size is shown next to each curve.

If this model were correct, then an increase in the number of SBF sites in the cell would increase the requirement for Cln3, and so would cause an increase in cell size at Start. We transformed otherwise wild-type cells with a high copy number plasmid containing four tandem, perfect SBF binding sites (an SBF binding site is called an “SCB”). Since the plasmid has a copy number of about 30, this provides about 120 extra sites, or roughly a 20% increase over the wild-type number of sites. We used elutriation to collect small G1 phase cells carrying the 4×SCB plasmid (or a control plasmid lacking the 4×SCB insert), then let these cells grow. We assayed cell volume, and the percentage of budded cells as an assay of Start. As shown in Figure 7, cells lacking the 4×SCB insert went through Start at about 32 fl, while cells containing the 4×SCB insert went through Start at about 38 fl, roughly a 20% increase. This experiment is consistent with the idea that the cell is setting the critical size for Start by titrating some molecule against the number of available SBF binding sites.

This experiment was done using several independent pairs of transformants a total of five times (i.e., five pair-wise comparisons). In every case, the strain with the 4×SCB plasmid had a larger critical size than the strain with the control plasmid. The differences in critical size in the five experiments were 2.6, 3.7, 6.1, 9.2, and 10.6 fl, with a mean of 6.4 fl (*p*<0.005 for a test of the hypothesis that the difference is 0 using a paired sample one-tailed Student's *t*-test; also statistically significant by nonparametric tests). The experiment shown in Figure 7 is the median experiment, with a 6.1 fl difference.

One issue with this titration experiment is that the 4×SCB plasmid might increase critical size through some irrelevant pathology. If this were so, then the 4×SCB plasmid would cause roughly the same percentage increase in size regardless of any changes we might make to the *CLN3/WHI5*/SBF system. A second issue is that even if the activator titration model is correct, it is not clear what activator is being titrated; it might be Cln3, but Swi4, Swi6, and even Stb1 are also possibilities.

To address these issues, we first repeated the experiment in a strain carrying two copies of *CLN3* (2×*CLN3*; a second copy is tandemly integrated at the wild-type *CLN3* locus). If the titration hypothesis is correct, then the second copy of *CLN3* should largely compensate for the ∼20% increase in SCB sites. Indeed, the increased size caused by the 4×SCB plasmid in a 2×*CLN3* background is only 0.6 fl (Figure 7). A 2×*CLN3* 4×SCB strain had almost exactly the same size as a wild-type (i.e., 1×*CLN3*) strain bearing the control plasmid. (We note that a second copy of *CLN3* causes only ∼10% decrease in critical size in a wild-type strain [38]. Presumably when Cln3 is sufficiently abundant, some other molecule becomes limiting for Start.)

In addition, we did the titration experiment in a *cln3* deletion strain. If the effect of the 4×SCB plasmid is irrelevant pathology, then in the *cln3* strain, the same irrelevant pathology should occur, and the 4×SCB plasmid should again increase critical size. On the other hand, if Cln3 is the activator being titrated, then in the *cln3* strain, the 4×SCB plasmid should have no effect on critical size, since it has nothing to titrate. In fact, to our great surprise, we got neither of these results. Instead, *cln3* cells actually got smaller when we added the 4×SCB plasmid (Figure 7). This surprising result was confirmed with two additional experiments (unpublished data), using independently constructed strains. (The experiment shown has the median difference of the three experiments.) This result tells us two things: first, the results are not irrelevant pathology, because the results change in a specific way with changes in the *CLN3/WHI5*/SBF system. Second, a likely interpretation is that Cln3 is indeed the activator being titrated (otherwise a deletion of *CLN3* would make no difference), but that the 4×SCB plasmid is also titrating repressors. That is, Cln3 is most limiting (so high copy 4×SCB causes bigger cells in a wild-type background), and repressors are next most limiting (so when there is no Cln3 anyway, then the high copy 4×SCB plasmid decreases size by titrating repressors).

If this is true, then a strain that lacks both Cln3 and also the repressors (Whi5 and Stb1) should not be affected by high copy 4×SCB. And this proves to be the case (Figure 7, bottom right); a *whi5 stb1 cln3* strain is not affected by the high copy 4×SCB plasmid. Note that the *whi5 stb1* mutant is not responsive to *CLN3* (Figure 1); the fact that it is also not responsive to extra SCBs is the expectation from the titration model.

The *whi5 stb1 cln3* strains shown in Figure 7 lack all known regulation of the Cln3 size control pathway. Yet, these strains have a size at budding similar to that of wild-type, and have a sigmoidal budding curve suggesting a dependence of budding on size. That is, although the best-characterized size control mechanism is missing, the cells apparently exhibit some form of size control. This suggests the existence of a redundant size control mechanism. The same phenomenon has been observed previously in different circumstances [39], where the redundant size control was attributed to a translational mechanism. In addition, Jorgensen et al. [22] found many size control mutants that were not in the *CLN3* pathway.

## Discussion

Here we have found that the Whi5 pathway is not the sole link between Cln3-Cdc28 and SBF activity. We have found several mutants that, like *whi5*, relieve the repression of SBF, and render its activity somewhat independent of Cln3-Cdc28. These mutants include *chd1*, *hda2*, *pho23*, *sin3*, *rpd3*, and *stb1*. Of these, *pho23*, *sin3*, *stb1*, and *rpd3*, are members of, or have been physically linked to, the Rpd3 histone deacetylase complex, a repressive histone deacetylase orthologous to mammalian HDAC1.

Although we do not know the exact relationship between these proteins and Whi5, we have found that the *stb1* mutation is synergistic with *whi5*; that is, in the context of a *bck2* mutation, the *stb1 whi5* double mutant, unlike either single mutant, has little ability to respond to Cln3-Cdc28. Thus in some sense Stb1 identifies a pathway for regulating SBF that is separate from the Whi5 pathway.

While we have identified *STB1* in a screen for repressors of SBF, others have previously identified *STB1* as an activator of SBF or MBF [33],[34]. While paradoxical at first sight, it is quite common for transcription factors to have both positive and negative roles in transcription. An example is Fkh2, which collaborates with Mcm1 and Ndd1 transcription factors, and with Clb-Cdc28 kinase activity, to regulate mitotic genes. In this context, Fkh2 appears to be an activator in late G2 and mitosis, but a repressor at other times [40]–[44]. Similarly, we imagine that Stb1 helps repress SBF in the absence of Cln3 and Bck2 (the situation in which we found it as a repressor), but helps activate SBF in the presence of Cln3 or Bck2 (the situation in which it was characterized as an activator). Consistent with this, cell cycle expression analysis of *stb1* mutants shows that target genes are less repressed at troughs, and less induced at peaks; i.e., they are less regulated and more constitutive (e.g., Figure 3 in [34]). The fact that *CLN3* can induce expression of *CLN2* even before Sin3, Rpd3, and Whi5 are lost from the promoter (Figure 4) is consistent with the idea that the initial expression of *CLN2* depends on activation, perhaps via Stb1, rather than on loss of repression.

If Stb1 is both a repressor and an activator, then some of our assays may preferentially see one of these activities, and some may see the other. Presumably it is the lack of repression by Stb1 that allows the *stb1* mutation to suppress the lethality of the *cln3 bck2* mutant. But the cell size assay for responsiveness to *CLN3* (Figure 1) may be more sensitive to Stb1 as an activator; in particular, the synergistic defect between *whi5* and *stb1* may be due to the lack of repression in the *whi5* mutant, plus the lack of activation in the *stb1* mutant. We note that the combinations of mutations that include *stb1* tend to have relatively large cell sizes after induction of *GAL-CLN3* (Figure 1), perhaps showing that *STB1* is needed for full induction of *CLN2*.

**Figure 8:**
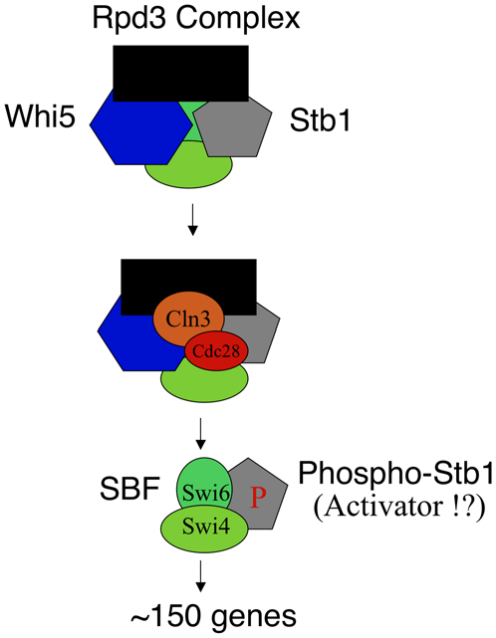
Model for regulation by Whi5, Stb1, and histone deacetylase. The SBF complex (green) recruits Whi5 (blue) and Stb1 (grey). The SBF complex also recruits the repressive Rpd3 histone deacetylase complex (black); this recruitment may be aided by Whi5 and/or Stb1. In a growth- and size-dependent way, the Cln3-Cdc28 kinase (orange-red) is recruited. This CDK phosphorylates Whi5 and Stb1 and possibly Swi6, resulting in the loss of the Rpd3 complex and the loss of Whi5. The transcriptional activation domain of Swi6 is revealed, possibly aided by an activating function of phospho-Stb1.

While Whi5 and Stb1 seem to define two pathways of regulation of SBF, it is still unclear how the Sin3-Rpd3 histone deacteylase complex is recruited to the *CLN2* promoter. Previously, the Rpd3 complex has been linked to Stb1 [29],[31]. More recently, Huang, Kaluarchchi and Andrews have found evidence for an association between Rpd3 and Whi5 (personal communication). Despite these associations, we found that even *whi5 stb1* double mutants had at least some Sin3 (and so presumably Rpd3) at the *CLN2* promoter, whereas *swi6* mutants had little or no Sin3. Thus although one could imagine various relationships between these proteins, one model is that SBF has some ability to recruit each of Whi5, Stb1, and Sin3-Rpd3, but that these proteins in addition interact with each other (Figure 8). Later, in a size- and growth-dependent fashion, Cln3-Cdc28 also joins the complex, and phosphorylates Whi5 and Stb1 and probably Swi6 and possibly Swi4. This causes the loss of the Rpd3 complex; a somewhat slower loss of Whi5 (Figure 4); and perhaps allows phosphorylated Stb1 to help activate transcription (Figure 8). That Swi6, along with Whi5 and Stb1, is probably a target of Cln3-Cdc28 phosphorylation is strongly suggested by the fact that over-expression of a mutant Whi5 lacking CDK phosphorylation sites is lethal in a mutant where Swi6 is likewise lacking CDK phosphorylation sites [20],[45]. The involvement of Swi6 as a likely target of Cln3-Cdc28, and as a recruiter of Sin3-Rpd3, may explain why even *whi5 stb1* double mutants seem to have some slight residual Cln3-responsiveness (Figure 1); that is, this residual responsiveness could be through direct phosphorylation of Swi6.

Results reminiscent of ours with regard to Sin3 and Rpd3 were previously obtained by Veis et al. [36], who found that Sin3 and Rpd3 associate with the promoter of the *CLB2* gene, which encodes a mitotic cyclin. Although *CLB2* is most highly expressed in G2/M, the association of Sin3 and Rpd3 with the *CLB2* promoter was lost in late G1, at about the same time we see loss of Sin3 and Rpd3 from the *CLN2* promoter. Veis et al. interpreted their results in terms of the association between Sin3/Rpd3 and the Fkh2 (forkhead) transcription factor, and suggested that this association was sensitive to Start. However, we note that *CLB2*, despite being most strongly up-regulated in G2/M, is a client of SBF as well as a client of Fkh2. The *CLB2* promoter contains at least three clustered SBF/MBF binding sites, at least two of which are conserved in other species of yeast [46]. In ChIP experiments, *CLB2* is a target of SBF or MBF binding [47],[48]. Thus the loss of Sin3/Rpd3 from the *CLB2* promoter in late G1 as seen by Veis and coworkers could involve SBF at the *CLB2* promoter, and so could be related to the phenomenon we see at the *CLN2* promoter.

Another protein we find at the *CLN2* promoter is Cln3. However, demonstrating this association was difficult, and required a special genetic background and over-expression of Cln3. Part of the difficulty in ChIPing Cln3 to the *CLN2* promoter is presumably because Cln3 is a nonabundant protein, and does not bind DNA directly. But in addition, Cln3 may not be a stoichiometric member of the complex. Instead, it may bind weakly and transiently, phosphorylate its substrate(s), and leave. The two proteins we find to be essential for Cln3 responsiveness, Whi5 and Stb1, are both very likely substrates of Cln-Cdc28 [20],[21],[34].

Cln3 is present at only about 100 molecules of protein per cell, and yet there are in the vicinity of 400 functional binding sites for SBF and the related factor MBF. The fact that Cln3 is sub-stoichiometric with respect to binding sites could provide a partial solution to the size control problem: Perhaps the amount of Cln3 in the cell, which is a function of cell size and growth rate, is titrated against the number of binding sites. And indeed we found that cells containing extra SCBs had to grow to a larger size to accomplish Start, and this effect could be compensated by one extra dose of *CLN3*. Extra SCBs did not enlarge a *cln3* null mutant, and extra SCBs had no effect whatever on *cln3 stb1 whi5* triple mutants. These findings are all supportive of the titration model.

Even though larger G1 cells contain more Cln3 molecules than smaller cells, the increase in Cln3 content with size is probably quite moderate, possibly only linearly correlated with cell size. Thus even at cell sizes adequate for Start, Cln3 may still be sub-stoichiometric with respect to binding sites. Thus we imagine that at any and all physiologically reasonable concentrations of Cln3, there will only be fractional occupancy of SBF sites, especially if Cln3 is a weak and transient binder. But as the amounts of Cln3 rise, and are titrated against a fixed number of SBF sites, that fractional occupancy will rise, until at some occupancy (i.e., at some critical cell size), *CLN2* and other targets are expressed, and the cell passes through Start. The issue is, how to convert a relatively small change in total Cln3 into a large change in fractional occupancy, or, alternatively, how to convert a small change in occupancy into a large effect?

Although we do not know the answers to either of these questions, Ferrell and coworkers have described many mechanisms by which such “super-sensitivity” can occur [49]–[56]. One mechanism would use the fact that SBF target genes have multiple SBF binding sites. Perhaps the binding of Cln3 to SBF is cooperative; or perhaps the Cln3 molecules, once bound, cooperate to do something else, such as phosphorylate a substrate. Cooperativity of any kind between multiple sites will give exponential sensitivity to Cln3 amounts, so this is one possible mechanism. A second mechanism is multisite phosphorylation. That is, perhaps the substrates of Cln3-Cdc28 have to be phosphorylated at multiple sites, and this can only happen when fractional occupancy of SBF sites by Cln3 is relatively high. Since phosphorylation is in a dynamic equilibrium with dephosphorylation, a requirement for multisite phosphorylation (at, say, five sites) imposes a super-sensitive threshold on the amount of kinase required [52],[57],[58]. Multisite phosphorylation can give extreme sensitivity to the amounts of a protein kinase [52],[57],[58]. A third possible mechanism is to consider the relationship between the complexes at the multiple SBF sites. There are three sites at *CLN2*; if all three have repressive proteins, is enough Cln3 needed to fill all three sites simultaneously, even though occupancy of any one site is always transient? At any rate, although we do not know how supersensitivity works in this situation, there are lots of ways it could work in theory, as cited above.

There are remarkable parallels between the SBF/Cln3/Whi5,Stb1/Rpd3 regulatory module in yeast, and the E2F-Dp/Cyclin D1/Rb/HDAC1 regulatory module in mammalian cells. To begin with, the cluster of regulated genes is highly conserved: In *S. cerevisiae* [6], in the distantly related yeast *S. pombe* [59], in mammalian cells [60], and probably in most or all other eukaryotes, there is a highly conserved cluster of genes needed for DNA replication, and expressed around the G1/S transition. In both yeasts and mammals, the motifs regulating these genes contain a core “CGCG” element. In both yeasts and mammals, the transcription factors recognizing this element (SBF/MBF in the yeasts, E2F-Dp in mammals) contain a DNA binding domain with a “winged helix” fold [61]–[63]. There is no apparent sequence homology between the yeast and mammalian DNA binding domains, but the domain is small, the evolutionary distance vast, and there are other examples where structure but not sequence has been preserved across time.

In E2F-Dp, the transactivator is repressed by binding of Rb and its family members. There are two mechanisms of repression [64]–[70]. First, the transactivation domain is masked. Second, Rb family members (but possibly not Rb itself-[69]) recruit mSin3B and HDAC1 which deacetylate and otherwise modify chromatin so as to be inhospitable towards expression. Here, we likewise show that there are at least two pathways of regulation, one of them involving recruitment of a histone deacetylase. In mammals, the transactivation domain is unmasked when a cyclin-CDK complex such as cyclin D-CDK4 phosphorylates Rb and family members, disrupting binding to E2F-Dp, and allowing Sin3m and HDAC1 to leave the chromatin. Similarly, in yeast, Cln3-CDK phosphorylates Whi5 and probably Stb1. Whi5, Sin3, and Rpd3 all leave the chromatin. Interestingly, expression of the target gene Cln2 precedes the loss of the repressive proteins, consistent with a dominant activation, possibly due to Stb1. In any case, it is clear that there are deep, well-conserved parallels between the SBF/Cln3/Whi5,Stb1/Rpd3 regulatory module in yeast, and the E2F-Dp/Cyclin D1/Rb/HDAC1 regulatory module in mammals. It is possible that these modules have regulated the cluster of genes for DNA synthesis since early in eukaryotic evolution.

## Materials and Methods

### Strains

Strains are shown in Table 2.

**Table 2:** Plasmids and strains.

Plasmid or Strain	Name	Genotype	Source
Plasmids	pBA70s	*CYC1::lacZ* 2 micron *URA3*	M. Tyers/B. Andrews
	pMT3579	*SCB-lacZ* 2 micron *URA3* (“pSCB”)	M. Tyers/B. Andrews
	pCM273	*TETp-CLN3-6FLAG CEN URA3*	M. Aldea
Strains	HWL49 background		
	HWL49	*MATα leu2 ura3 trp1 his3 GAL-CLN3::URA3 bck2::TRP1 cdc34-2*	H. Wang
	HWL51	*GAL-CLN3::URA3 bck2::TRP1 cdc34-2 SIN3-TAP*	
	HWL59	*GAL-CLN3::URA3 bck2::TRP1 cdc34-2 RPO21-TAP*	
	HWL61	*GAL-CLN3::URA3 bck2::TRP1 cdc34-2 SWI4-TAP*	
	HWL62	*GAL-CLN3::URA3 bck2::TRP1 cdc34-2 RPD3-TAP*	
	HWL63	*GAL-CLN3-TAP::KanMX bck2::TRP1 cdc34-2*	
	HWL77	*GAL-CLN3::URA3 bck2::TRP1 cdc34-2 WHI5-TAP*	
	HWL128	*GAL-CLN3::URA3 bck2::TRP1 cdc34-2 STB1-TAP*	
	BF305-15d background		
	BF305-15d	*MATa leu2 his3 ura3 trp1 ade1 arg5,6 met14*	B. Futcher
	HWL145	*cln3::LEU2*	
	HWL146	*cln3::LEU2 whi5::KanMX stb1::NAT1*	
	HWL148	*CLN3::URA3::CLN3*	
	S288c background		
	S288c	*MAT*α *mal gal2 flo8*	R. Mortimer
	HWL99	*SIN3-TAP::KanMX whi5::NAT1*	
	HWL110	*SIN3-TAP::KanMX*	
	HWL117	*WHI5-TAP sin3::NAT1*	
	HWL119	*SIN3-TAP stb1::NAT1*	
	W303 background		
	W303-1a	*MATa ade2 trp1 leu2 his3 ura3 can1-100 {psi+}*	R. Rothstein
	HWL72	*tTA::LEU2*	
	HWL112	*SWI6-13MYC::KanMX4 tTA::LEU2*	
	HWL130	*SWI6-13MYC::KanMX4 swi4::NAT1 tTA*::*LEU2*	
	N497	*cln3*::*ura3 bck1*::*TRP1 rme1*::*hisG*	N. Edgington
	N499	*cln3*::*ura3 bck1*::*TRP1 rme1*::hisG *whi5*::KanMX	
	N451	*cln3*::*ura3 bck1*::*TRP1 rme1*::hisG *stb1*::KanMX	
	N452	*cln3*::*ura3 bck1*::*TRP1 rme1*::hisG *sin3*::KanMX	
	N453	*cln3*::*ura3 bck1*::*TRP1 rme1*::hisG *rpd3*::KanMX	
	N454	*cln3*::*ura3 bck1*::*TRP1 rme1*::hisG *hos3*::*LEU2*	
	BY4701/4702 background		
	OBS1	*MATa his3 leu2 ura3 met15 WHI5-TAP*	Open Biosystems
	OBS5	*MATa his3 leu2 ura3 met15 STB1-TAP*	Open Biosystems
	LC504	*URA3-GAL-CLN*3 *bck2::KanMX stb1::KanMX whi5::KanMX*	L. Carey
	LC517	*URA3-GAL-CLN3 bck2::KanMX*	
	LC518	*URA3-GAL-CLN3 bck2::KanMX stb1*::*LEU2*	
	LC520	*URA3-GAL-CLN3 bck2*::*KanMX*::*HIS3 whi5*::*LEU2*	
	LC521	*URA3-GAL-CLN3 bck2*::*KanMX*::*HIS3 whi5*::*LEU2 rpd3*::*KanMX*	
	LC523	*URA3-GAL-CLN3 bck2*::*KanMX*::*HIS3 whi5*::*LEU2 hda2*::*KanMX*	
	LC524	*URA3-GAL-CLN3 bck2*::*KanMX*::*HIS3 whi5*::*LEU2 sin3*::*KanMX*	

Strains were created during this study unless otherwise indicated. LC strains are from crosses between BY4741 and BY4742 strains. They are *his3 leu2 ura3* with *MAT*, *met15* and *lys2* segregating.

### Cell Cycle Synchronization

For *GAL-CLN3 cdc34-2* block and release experiments, cells growing in YEP with 2% raffinose+2% galactose (YEPRG) at 25°C were arrested in G1 by washing with YEP+2% raffinose (YEPR) and incubating in YEPR for four hours at 25°C. Cells were then shifted to 37°C for 1 h, and then cultures were split in two; one half remained in YEPR and to the other half galactose was added to 2% final concentration. Both cultures were incubated at 37°C and samples were taken every 5 min.

For elutriations, cells containing plasmids (pBA70 and pMT3579) were grown in Synthetic Complete (SC) medium with 2% filter-sterilized sucrose as the carbon source. Small unbudded G1 cells were isolated by centrifugal elutriation and grown in preconditioned SC+2% sucrose at 30°C. Cell size distributions were obtained on a Z2 Coulter Counter and budding indexes were determined by counting cells. Bud counts were done “blind” on randomized samples.

### Immunoprecipitations, Northern, and Western blots

Immunoprecipitations and Northern and Western blots were carried out essentially as described previously [71],[72]. Cell lysates were obtained by vortexing cell suspensions in lysis buffer (0.1% NP40, 250 mM NaCl, 50 mM NaF, 5 mM EDTA, and 50 mM Tris [pH 7.5]) in the presence of glass beads. Cell debris was pelleted by centrifugation for 10 min at 4°C. Protein concentration in the lysate was determined by the DC protein assay (Bio-Rad). Lysates typically contained 20–50 mg/ml of total protein. For immunoprecipitation, 5 mg of cell lysate mixed with 2 µl of 9E10 anti-Myc ascites fluid or 5 µg of anti FLAG antibody (Sigma) were rotated for 1–2 h at 4°C. Immune complexes were collected on protein A-Sepharose beads by rocking at 4°C for 1 h. For detection of immunoprecipitated proteins, beads were pelleted by very gentle, brief, low-speed centrifugation, washed four times with lysis buffer, and boiled in protein sample buffer immediately before SDS-PAGE.

### ChIPs

Early exponential phase cells were collected and formaldehyde was added to 1% final concentration. Cells were fixed at room temperature for 15 min. Cross-linking was quenched by the addition of glycine to 125 mM. Cells were pelleted at 3,000 *g* for 5 min and washed twice with ice-cold TBS (150 mM NaCl, 20 mM Tris-HCl [pH 7.6]). To break cells, cell suspensions in lysis buffer (50 mM HEPES-KOH [pH7.5], 140 mM NaCl, 1 mM EDTA, 1% Triton X-100, 0.1% sodium deoxycholate) were mixed with glass beads and vortexed at 4°C for 45 min. Chromatin was sheared by sonication at power 3 (W-380 Sonicator, Heat Systems-Ulrasonic, INC) ten times, 10 s each time, and tubes were kept on ice throughout sonication. Cell debris was removed by maximal speed centrifugation for 15 min at 4°C. Whole-cell extracts were prepared for use in ChIPs. Protein concentration for each sample was determined by DC protein assay (Bio-Rad). Immunoprecipitations were performed with 1 mg of extract. Lysates were rotated with 25 µl IgG Sepharose beads at 4°C overnight. Immune complex beads were washed with lysis buffer, lysis buffer 500 (50 mM HEPES-KOH [pH 7.5], 500 mM NaCl, 1 mM EDTA, 1% Triton X-100, 0.1% sodium deoxycholate), and LiCl/detergent (0.5% sodium deoxycholate, 1 mM EDTA, 250 mM LiCl, 0.5% NP-40, 10 mM Tris [pH 8.0]) twice for each buffer and washed once with cold TE. Bead washing was performed at 4°C. DNA was eluted by incubating beads at 65°C with elution buffer (10 mM EDTA, 1% SDS, 50 mM Tris.Cl [pH 8.0]) for 10 min, and crosslinks were reversed by incubating samples at 65°C overnight. PCR was carried out for 30 cycles and products were separated using 2.4% agarose gels.

### qReal Time-PCR

A SuperScript III Platinum SYBR green one-step q(real-time) RT-PCR kit (Invitrogen) was used for the detection and quantification of RNA. 5 ng RNA was used for the RT-PCR reaction. Total RNA were purified with RiboPure-Yeast kit (Ambion).

### Asynchronous Cell Size

Cells were grown overnight in either YEPR or YEPRG so that cell densities were between 1 and 2×10^7^ cells/ml. Cultures were placed on ice, sonicated to separate mothers from daughters, and cell sizes were measured on a Z2 Coulter Counter. Cells were then photographed at 40× and brightness and contrast adjusted in Adobe Photoshop. All data shown are from cells in the S288c genetic background; cells in both the W303 and BF305 backgrounds were also tested, and gave identical results.

### Genetic Suppression

Cells were grown overnight in SC-Met, sonicated briefly, and 1∶4 serial dilutions were plated onto either SC-Met or SC+2 mM Met plates. Cells on SC-Met plates were grown for 3 d at 27°C before being photographed, whereas cells grown on SC+2 mM Met plates were grown for 5 d at 27°C.

## Abbreviations

CDK: cyclin dependent kinase
ChIP: chromatin immunoprecipitation
qPCR: quantitative PCR
